# The early predictive role of complement C3 and C4 in patients with acute pancreatitis

**DOI:** 10.1002/jcla.23205

**Published:** 2020-03-18

**Authors:** Lifeng Zhang, Zhenguo Qiao, Huang Feng, Jiaqing Shen

**Affiliations:** ^1^ Department of General Surgery The First Affiliated Hospital of Soochow University Suzhou China; ^2^ Department of Gastroenterology Affiliated Wujiang Hospital of Nantong University Suzhou China; ^3^ Department of Gastroenterology The First Affiliated Hospital of Soochow University Suzhou China

**Keywords:** acute pancreatitis, complement C3 and C4, peripheral blood, prediction, severity

## Abstract

**Objective:**

The prognostic role of complement C3 and C4 in peripheral blood in early stage of acute pancreatitis (AP) is unknown. In this study, we aimed to evaluate the prognostic value of C3 and C4 in early stage of AP.

**Methods:**

A total of 164 patients were enrolled in this study. The blood samples were collected within 24 hours after AP onset. We compared C3 and C4 levels in patients with different AP severity. The optimal cutoff value for them to predict severe AP (SAP) was determined by receiver operating characteristic (ROC) curve analysis.

**Results:**

The reduction of C3 and C4 levels was observed. For prediction of MSAP and SAP, the AUC of C3 and C4 levels was 0.695 (95% CI: 0.612‐0.779) and 0.739 (95% CI: 0.657‐0.821). The cutoff value of C3 and C4 levels was 0.705 and 0.145 g/L, with the sensitivity of 0.612 and 0.735, and the specificity of 0.735 and 0.710. For prediction of SAP, the AUC of C3 and C4 levels was 0.749 (95% CI: 0.607‐0.891) and 0.766 (95% CI: 0.596‐0.936). The cutoff value of C3 and C4 levels was 0.400 and 0.125 g/L, with the sensitivity of 0.859 and 0.767, and the specificity of 0.600 and 0.786.

**Conclusions:**

A marked change of complement C3 and C4 was observed in peripheral blood of patients with AP, suggesting the participation of complement system in the early phase of AP. C3 and C4 levels were sensitive and accurate in judging the severity of AP.

## 1 INTRODUCTION

Acute pancreatitis (AP) is a common disease in the department of gastroenterology. The main etiologies of AP are biliary tract diseases, alcohol over‐consumption, and hyperlipidemia.^1^ Most of AP patients involve regional inflammation and/or necrosis of the pancreas which will result in the discharge of a variety of inflammatory mediators. However, a small proportion of patients will suffer from the systemic inflammatory reaction caused by the over‐production of these inflammatory mediators.^2^ For those patients, which are thought as severe AP (SAP), systemic inflammatory response syndrome and various organ dysfunctions are finally induced secondary to the pancreatic damage.^3^ In SAP, high morbidity and mortality are often observed clinically.^4^, ^5^ Thus, the early and effective assessment of severe cases is still of urgency and benefits the early interventions.

Immunologic impairment in the early phase of AP is linked to the increased susceptibility to subsequent infectious complications such as infected pancreatic necrosis and sepsis,^6^ which are the most serious complications in SAP and contribute to the mortality.^7^ As a part of the innate immune system, the systemic activation of complement plays an important role in chemotaxis and leukocyte activation, which represents the very early phenomenon in AP.^8^, ^9^, ^10^ Which is also thought to participate in the systemic inflammation and organ failure.^11^ In a clinical setting, the significantly attenuated levels of central complement components of the classical (eg, C1q and C4) and the alternative (eg, C3) pathways in plasma were detected in patients with SAP.^12^ As indicated by the consumption of plasma complement proteins, the intrapancreatic activation of the complement cascade may exert detrimental effects by adverse outcome in SAP.^13^ The involvement of complement in the pathogenesis of SAP is also revealed by results of animal experiments.^14^ However, the diagnostic potential of complement in peripheral blood remains unknown in the prediction of SAP. In the present study, we aimed to evaluate the role of complement components (C3 and C4) in judging the severity of AP.

## 2 MATERIALS AND METHODS

### 2.1 Patients

This study was approved by Institutional Review Board of The First Affiliated Hospital of Soochow University. Before the study, the written informed consent was given by all participants. Haitai Database (Nanjing Haitai Information Technology Co., Ltd.) was used to search the data of AP patients between January 2015 and December 2016. The diagnosis of AP was based on two of the following three features: (a) acute abdominal pain; (b) serum lipase or amylase activity is threefold more than the upper limit of normal value; and (c) characteristic changes in imaging.^15^ Imaging changes were tested again in all cases before discharge in order to determine the existence of the late‐stage complications. Exclusion criteria were as follows: (a) chronic cardiac and pulmonary diseases; (b) previous history of pancreatic diseases, including AP, chronic pancreatitis, and pancreatic cancer; (c) chronic renal failure; (d) chronic liver dysfunction; and (e) a history of malignancy.

### 2.2 Definition of the severity of acute pancreatitis

Three degrees of severity of AP were defined by the 2012 revision of the Atlanta classification and definitions by international consensus. Mild acute pancreatitis (MAP) was defined as a diagnosed AP without organ failure and local or systemic complications. Moderately severe acute pancreatitis (MSAP) was defined as a diagnosed AP accompanied with local or systemic complications without persistent organ failure (<48 hours). SAP was defined as a diagnosed AP accompanied with local or systemic complications with persistent organ failure (>48 hours).^15^

### 2.3 Definition of local complications

Local complications were comprised of acute peripancreatic fluid collection, pancreatic pseudocyst, acute necrotic collection, walled‐off necrosis, gastric outlet dysfunction, splenic and portal vein thrombosis, and colonic necrosis.^16^

### 2.4 Definition of organ failure

Organ failure was considered to exist when one of the following manifestations occurred: (a) shock (systolic pressure < 90 mm Hg); (b) pulmonary insufficiency (PaO_2_ ≤ 60 mm Hg); (c) renal failure (serum creatinine > 2.0 mg/dL after hydration); (d) gastrointestinal bleeding (>500 mL/24 h); (e) disseminated intravascular coagulation (platelets ≤ 100 000/mm^3^, fibrinogen ≤ 100 mg/dL, and fibrin split products > 80 mg/mL); (f) a severe metabolic disturbance (serum calcium ≤ 7.5 mg/dL); and (g) the presence of SIRS, which was defined by presence of two or more following criteria: (a) heart rate > 90 beats/min, (b) core temperature < 36 or >38°C, (c) white blood count < 4000 or >12 000/mm^3^, and (d) respirations > 20/min.^16^

### 2.5 Measurement of complement C3 and C4

The blood samples were collected within 24 hours after AP onset for the measurement of complement C3 and C4. Assays for complement C3 and C4 in serum of patients were conducted using a Human Complement C3 and Complement C4 Multiplex EFSIA kit (Beijing 4A Biotech Co., Ltd) according to the manufacturer's protocol.

### 2.6 Statistical analysis

Continuous variables were expressed as means (±SD) and categorical variables as absolute correlative frequencies and then analyzed using SPSS PC version 18.0 for Windows (SPSS Inc). The criterion of statistical significance was *P*‐values of <.05. The significances of differences between the distributions of quantitative variables were assessed by using the Student's *t* test and qualitative variables by using the chi‐square test. Non‐parametric data were assessed using the Whitney *U* test. The area under the receiver operating characteristic (ROC) curve was used to assess the predictive accuracy.

## 3 RESULTS

**Figure 1:**
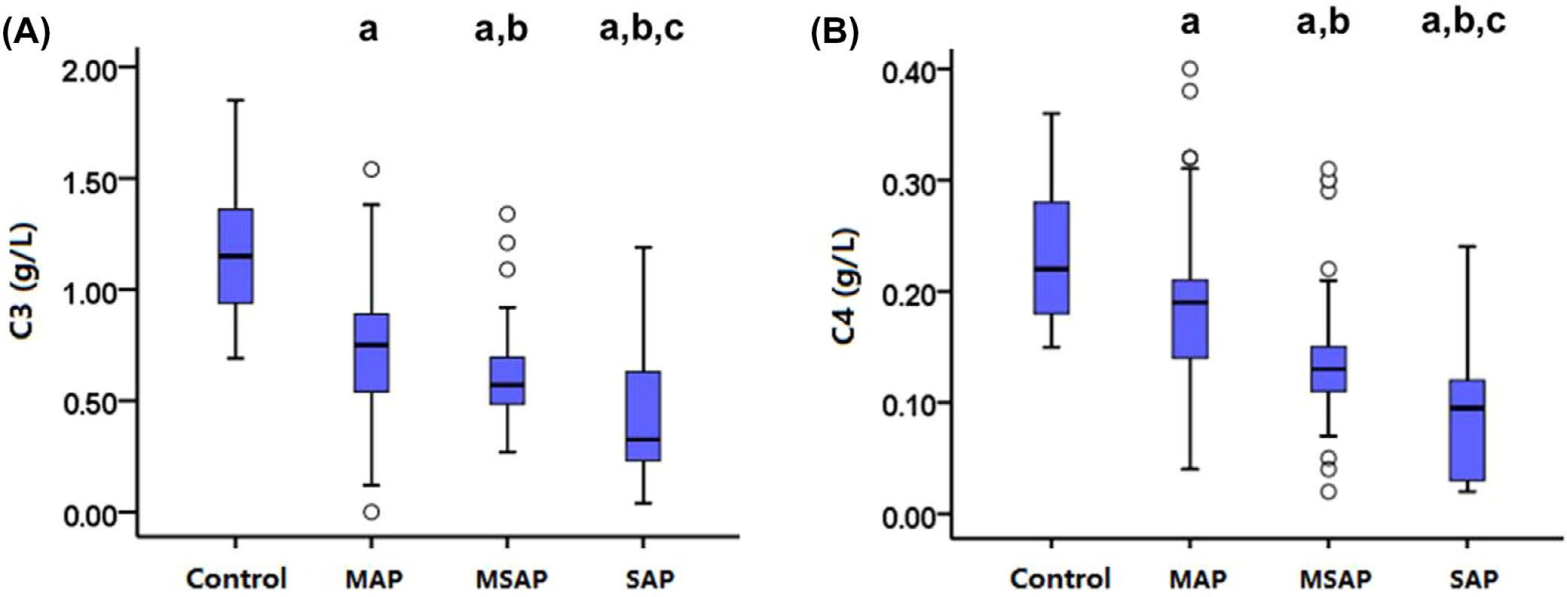
Comparisons of C3 and C4 in control, MAP, MSAP and SAP in the early stage of AP. ^a^ *P* < .05, compared with control; ^b^ *P* < .05, compared with MAP; ^c^ *P* < .05, compared with MSAP. MAP: mild acute pancreatitis; MSAP: moderately severe acute pancreatitis; and SAP: severe acute pancreatitis

**Figure 2:**
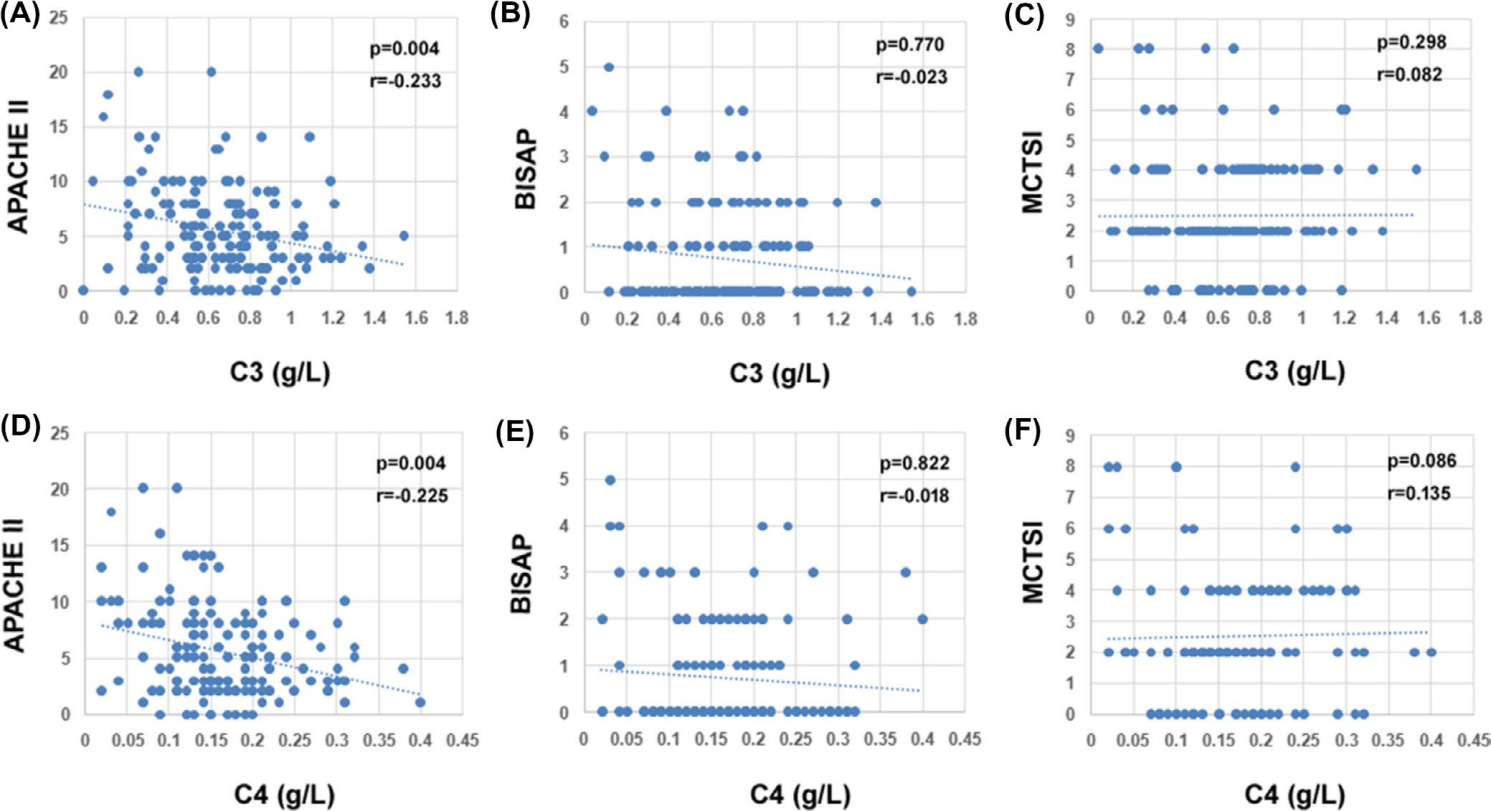
The correlation between C3, C4 and APCHE II, BISAP and MCTSI scores. The correlation between C3, C4, and APACHE II scores was also observed (C3: *r* = −.233, *P* = .004; C4: *r* = −.225, *P* = .004). The APACHE II scores reduced along with the increase of C3 and C4 levels

A total of 164 patients were enrolled in this study. A total of 103 patients were finally diagnosed as MAP, 47 as MSAP and 14 as SAP. A total of 22 healthy people were served as control. Baseline characteristics of these patients were presented in Table 1. The concentration of C3 and C4 reduced with the increase of the severity of AP(C3 [g/L]: Control: 1.16 ± 0.29; MAP: 0.72 ± 0.28; MSAP: 0.61 ± 0.22; SAP: 0.42 ± 0.31; C4 [g/L]: Control: 0.23 ± 0.06; MAP: 0.19 ± 0.07; MSAP: 0.14 ± 0.06; SAP: 0.10 ± 0.08) (Figure 1).A negative correlation between C3 levels and APACHE II scores was observed (C3: *r* = −.233, *P* = .004). A similar tendency was also found in C4 levels (C4: *r* = −.225, *P* = .004) (Figure 2).

**Table 1:** Characteristics of enrolled AP patients

	Control (N = 22)	MAP (N = 103)	MSAP (N = 47)	SAP (N = 14)
Age (years)	47.45 ± 17.84	50.58 ± 16.21	53.87 ± 19.06	51.86 ± 18.88
Gender (M/F)	13/9	54/49	26/21	8/5
Etiology				
Biliary	‐	50	25	7
Hyperlipidemic	‐	24	8	5
Alcoholic	‐	13	6	2
Others	‐	16	8	0
WBC (×10^9^/L, <24h)	6.09 ± 1.13	8.44 ± 2.56^a^	12.52 ± 3.27^a^, ^b^	16.00 ± 3.70^a^, ^b^, ^c^
APACHEII scores	‐	3.89 ± 2.58	7.17 ± 4.20^b^	12.50 ± 3.37^b^, ^c^
BISAP scores	‐	1.18 ± 1.02	1.72 ± 0.99^b^	3.07 ± 1.00^b^, ^c^
MCTSI	‐	1.87 ± 1.43	2.33 ± 1.41^b^	6.00 ± 1.92^b^, ^c^
CRP (mg/L)	5.27 ± 4.70	75.68 ± 63.45^a^	159.97 ± 103.66^a^, ^b^	208.93 ± 87.46^a^, ^b^, ^c^

Note

Values were expressed in mean ± SD.

Abbreviations: APACHE, Acute Physiology and Chronic Health Evaluation; BISAP, the bedside index of severity in acute pancreatitis; BMI, body mass index; CRP, C‐reactive protein; MAP, mild acute pancreatitis; MCTSI, modified computed tomography severity index; MSAP, moderately severe acute pancreatitis; SAP, severe acute pancreatitis.

a *P* < .05, vs control.

b *P* < .05, vs MAP.

c *P* < .05, vs MSAP.

ROC analysis was utilized to assess the diagnostic performance of C3 and C4 levels in predicting the severity of AP. For prediction of MSAP and SAP vs MAP, the area under the curve (AUC) of C3 and C4 levels was 0.695 (95% confidence interval [95% CI]: 0.612‐0.779) and 0.739 (95% CI: 0.657‐0.821). ROC curve for CRP showed the AUC was 0.807 (95% CI: 0.737‐0.877) (Table 2). For prediction of SAP vs MAP and MSAP, the AUC of C3 and C4 levels was 0.749 (95% CI: 0.607‐0.891) and 0.766(95% CI: 0.596‐0.936). The AUC of CRP was 0.834 (95% CI: 0.714‐0.820) (Table 3).

**Table 2:** Predictive factors in MSAP and SAP vs MAP

Variables	Area	SE	*P* value	95% CI		Cutoff value	Sensitivity	Specificity
				Lower bound	Upper bound			
C3	0.695	0.043	.000	0.612	0.779	0.705	0.612	0.803
C4	0.739	0.042	.000	0.657	0.821	0.145	0.735	0.710
CRP	0.807	0.036	.000	0.737	0.877	99.15	0.820	0.689

Abbreviations: CI, confidential interval; CRP, C‐reactive protein; MAP, mild acute pancreatitis; MSAP, moderately severe acute pancreatitis; SAP, severe acute pancreatitis; SE, standard error.

**Table 3:** Predictive factors in MAP and MSAP vs SAP

Variables	Area	SE	*P* value	95% CI		Cutoffvalue	Sensitivity	Specificity
				Lower bound	Upper bound			
C3	0.749	0.073	.001	0.607	0.891	0.400	0.859	0.600
C4	0.766	0.087	.001	0.596	0.936	0.125	0.767	0.786
CRP	0.834	0.046	.000	0.743	0.925	166.00	0.714	0.820

Abbreviations: CI, confidential interval; CRP, C‐reactive protein; MAP, mild acute pancreatitis; MSAP, moderately severe acute pancreatitis; SAP, severe acute pancreatitis; SE, standard error.

## 4 DISCUSSION

Primarily determined by the presence and duration of organ failure, three degrees of clinical severity of AP were defined according to the revised Atlanta Classification in 2012.^15^ The early identification of SAP is still one of the most difficult aspects of the early interventions of AP. Patients with SAP benefit from the early identification by being able to take advantage from the early management in an intensive care unit.^17^ That is why many AP related researches have being focused on possible predictors, which can assess the severity of AP at an early time point. Efforts in identifying predictors of AP severity have been going on for decades, but we still do not have a perfect predictor.^18^, ^19^, ^20^ Many predictive methods have been developed and validated to monitoring clinical changes in AP patients, including many biomarkers, radiological and clinical scoring systems. Among them, Acute Physiology and Chronic Health Evaluation II (APACHE II), Ranson scoring system, and BISAP are most widely used, but not entirely successful because of their own limitations. Some inflammatory mediators, proven to be mainly of pathophysiological interest, has been shown to be useful for predicting the course of AP, but none of them has been incorporated into routine clinical use yet.^21^ Thus, an objective, accurate, fast, and simple method is still necessary for the early intervention of potential SAP.

The potential of complement proteins for prediction of a severe course of AP remains controversial. One report indicates that the measurement of complement components (eg, C3 and C4) or complement fragments (C3c) does not have any implications for the management of AP.^22^ In contrast, another study has shown a significant correlation between attenuated complement components (eg, C1q, C3, and C4) or elevated complement fragments (C3a, C5a) and AP severity.^23^ Regarding serum complement factors, serum C3 and C4 levels fall significantly in AP. Lowered serum C3 is thought to be an unfavorable prognostic sign for the course of SAP. As compared to edematous pancreatitis, C3 and C4 are also found significantly decreased in patients with necrotizing pancreatitis.^6^ Similar to previous reports, the reduction of C3 and C4 in peripheral blood was also found in AP in the present study. The reduction was obviously associated with the severity of AP, further suggesting the participation of C3 and C4 in the pathogenesis of AP. In this study, we also attempted to test the potential effect of plasma C3 and C4 levels on the prognosis of the severity of AP. For the prediction of MSAP and SAP, C4 had a higher sensitivity. But, C3 had a higher sensitivity for the prediction of SAP, compared with C4. Plasma C‐reactive protein (CRP) is one of the most widely used parameters and forms the part of many guidelines. In the present study, CRP was more sensitive for the prediction of MSAP and SAP, compared with C3 and C4. Conversely, C3 and C4 were more sensitive for the prediction of SAP in comparison with CRP.

In conclusion, a marked change of complement C3 and C4 was observed in peripheral blood of patients with AP, suggesting the participation of complement system in the early phase of AP. C3 and C4 levels were significant predictors in judging the severity of AP.

## CONFLICT OF INTEREST

No conflict of interest exists in this article.
